# A Patient With Erdheim-Chester Disease Presenting With Intestinal Obstruction as the Initial Symptom: A Case Report

**DOI:** 10.3389/fonc.2022.849578

**Published:** 2022-03-16

**Authors:** Xiuzhi Zhou, Duchang Zhai, Junlin Yang, Dai Shi, Kuan Lu, Wu Cai, Guohua Fan, Shenghong Ju

**Affiliations:** ^1^ Department of Radiology, The Second Affiliated Hospital of Soochow University, Suzhou, China; ^2^ Department of Radiology, Zhongda Hospital, Medical School of Southeast University, Nanjing, China

**Keywords:** Erdheim-Chester disease, non-Langerhans cell histiocytosis, intestinal obstruction, bone imaging, oncology

## Abstract

Erdheim-Chester disease (ECD) is a rare and systemic non-Langerhans cell histiocytosis. Recently, ECD was classified as an inflammatory medullary tumor that affects a diverse group of organ systems. The purpose of this report is to present the radiological features of this disease in a 51-year-old man with intestinal obstruction as the initial presentation. In this case, X-ray computed tomography (CT) and emission computed tomography (ECT) clearly showed lesions in various systems, especially in the skeletal images. The survival benefit of treatment with interferon α (IFN-α) and BRAF inhibitors is well established, while other treatments focus on symptom relief.

## Introduction

Erdheim-Chester disease (ECD) is a rare non-Langerhans cell histiocytosis. ECD is considered an inflammatory myeloid neoplasm whose pathogenesis is related to BRAF^V600E^ mutation or other mutations in the MAPK pathway (1). It is a multisystem disease involving all organs, and intestinal involvement is even rarer. Clinical investigations vary according to the different organs involved. From the pathological perspective, a large amount of foamy histiocytic infiltration is observed under the microscope. The clinical and radiographic findings vary, but we present a patient with this lesion that shows multiple sclerosing features with systemic hyperplasia of soft tissues around multiple organs on CT images and multiple areas of hyperactive bone metabolism on ECT images (2, 3).

## Case Report

**Figure 1:**
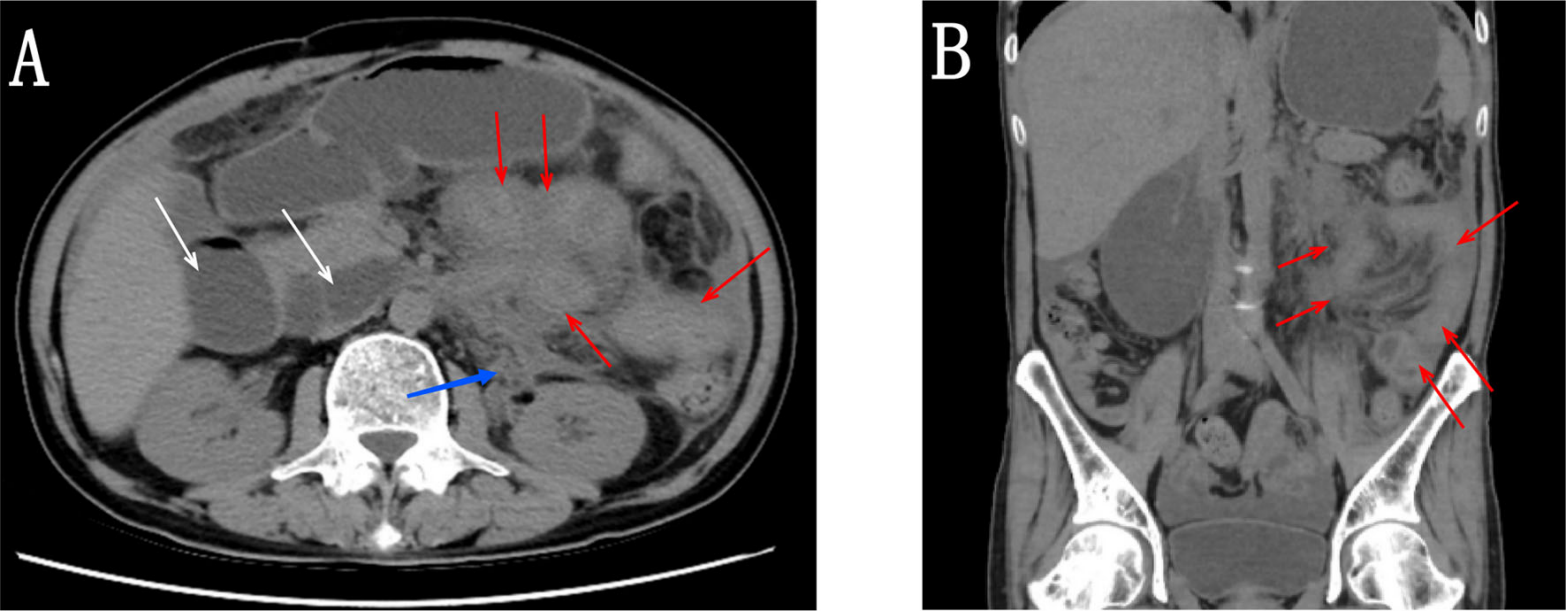
**(A, B)** Abdominal CT scan showing multiple areas with thickening of the intestinal wall (red arrow), soft tissue in the left perinephric space along the medial aspect (blue arrow), and dilation of intestinal lumen (white arrow).

A 51-year-old man was admitted to the hospital many times for recurrent intestinal obstruction. The patient reported a history of acid reflux and vomiting with a cessation of defecation 4 months previously, at which time an abdominal CT scan showed multiple areas with a thickening of the intestinal wall and dilation of intestinal lumen (**Figure 1**). At that time, the patient was diagnosed as proximal duodenal obstruction. This patient then received surgical treatment of enterolysis and duodenojejunal anastomosis and was discharged after improving. The pathology of this operation showed fibrocollagen hyperplasia accompanied by lymphocyte infiltration and histopathological infiltration. The patient was readmitted due to his poor appetite and vomiting three weeks later. The patient’s symptoms were alleviated after hormone therapy combined with anti-infective and nutritional support, and the patient was discharged after improving. The patient has been followed beginning after discharged, and to date, the patient has shown no recurrence of illness. Recently, an abdominal unenhanced CT scan revealed similar intestinal wall thickening and increased ascites. The patient had a history of syncopes and lost 15 kg in the last 2 years, initially weighing 60 kg. The patient underwent intracranial surgery for moyamoya disease nine months ago. The nutritional status of the patient was poor and scattered macular xanthelasmata was present in his skin.

**Figure 2:**
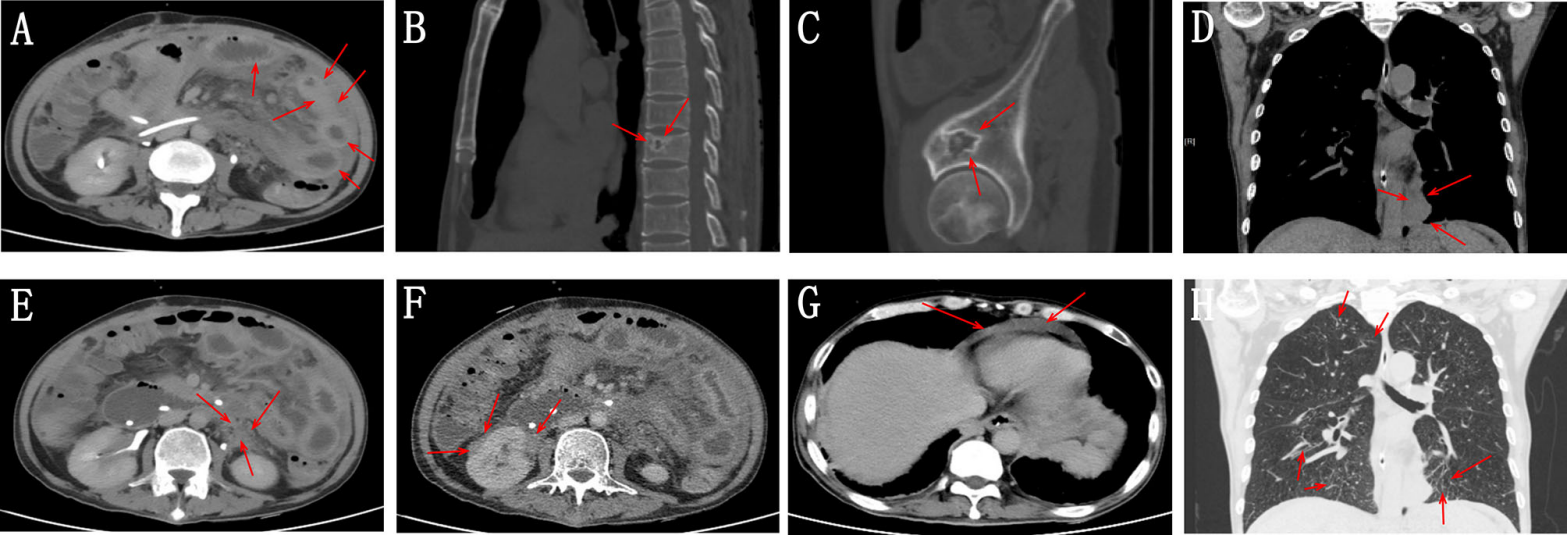
**(A)** Abdominal CT scan showing mural thickening of the small bowel loops (red arrow). **(B, C)** Whole-body CT scan showing lytic lesions with sclerotic margins in the vertebral bodies and iliac bones (red arrow). **(D)** Chest CT scan showing soft tissue in left paravertebral location (red arrow). **(E, F)** Abdominal CT scan showing retroperitoneum and perinephric soft tissue density foci (red arrow). **(G)** Chest CT image showing pericardial effusion. **(H)** Chest CT image showing reticular opacities in both lungs.

**Figure 3:**
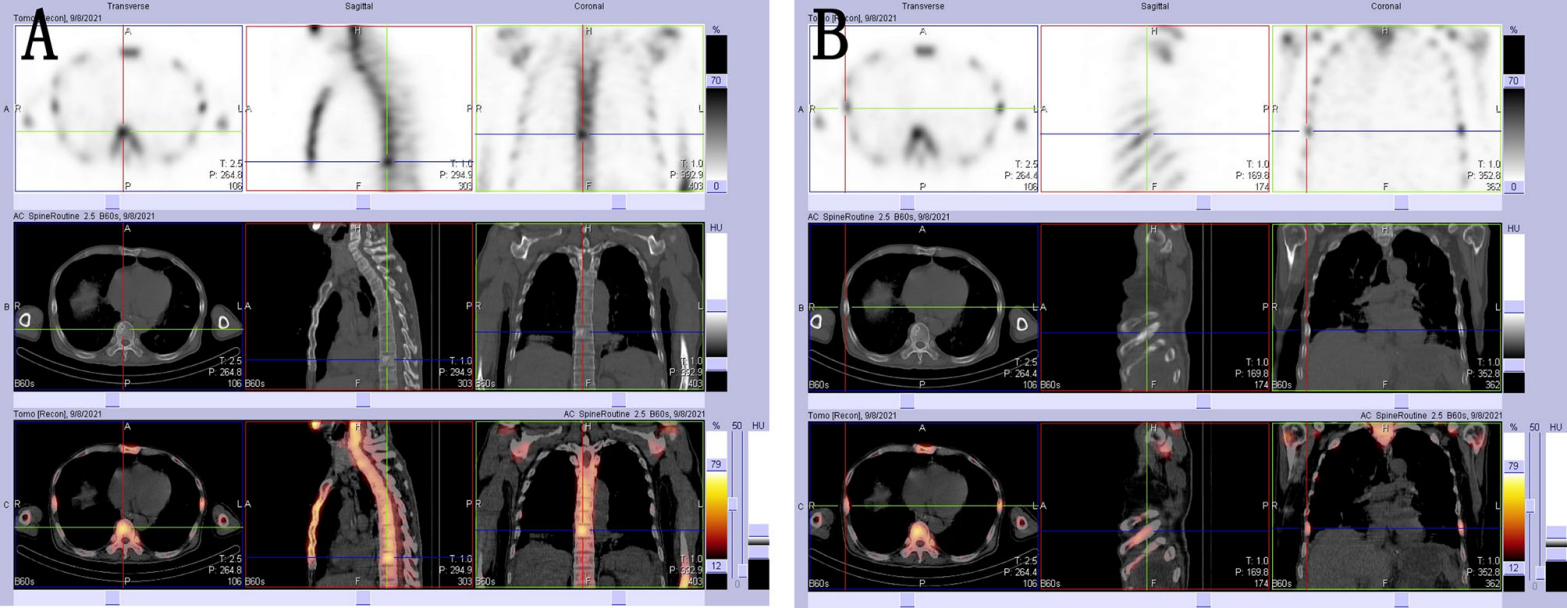
**(A, B)** 99mTc-MDP SPECT/CT images showing areas with high concentrations of radioactivity that were consistent with those observed on the CT scan.

**Figure 4:**
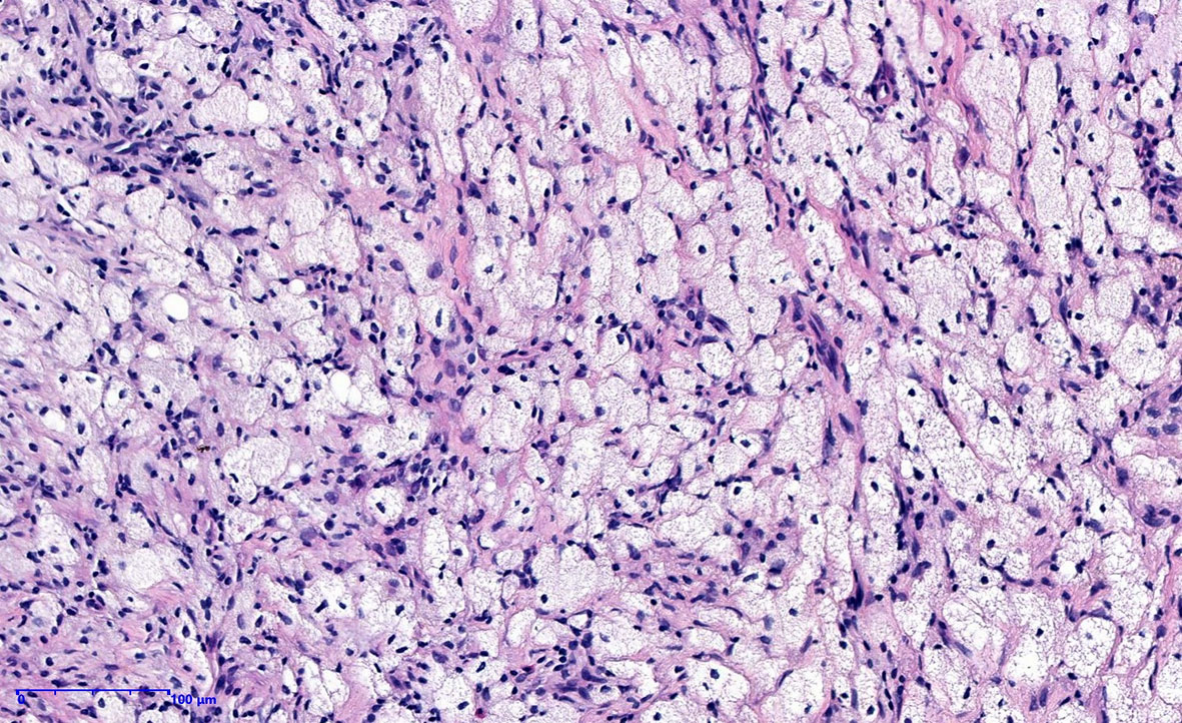
Histopathological image showing fibrocollagen hyperplasia with lymphocyte infiltration and foamy histiocytic hyperplasia.

The patient underwent a CT scan again to identify the cause of the recurrent intestinal obstruction. CT scan revealed mural thickening of the small bowel loops (**Figure 2A**), lytic lesions with sclerotic margins in the vertebral bodies and iliac bones (**Figures 2B, C**), soft tissue in left paravertebral location (**Figure 2D**), retroperitoneum (**Figure 2E**) and perinephric location (**Figure 2F**), pericardial effusion (**Figure 2G**), and reticular opacities in both lungs (**Figure 2H**). The patient also underwent a whole-body bone scan with technetium-99 to exclude the possibility of malignancy, which showed avid uptake in the bones (**Figures 3A, B**), suggesting possible manifestations of ECD. Low hemoglobin values and significantly elevated C-reactive proteins levels were detected using laboratory tests. The pathological examination of the small intestinal wall showed collagenous tissue hyperplasia with lymphocyte infiltration and foam cell infiltration (**Figure 4**). Immunohistochemical (IHC) analyses revealed that the cells were positive for CD68 but negative for the Langerhans tissue cell markers, AE1/AE3, CD1α and S100. After a multidepartment consultation, the patient was diagnosed with ECD. The patient received empiric hormone therapy and symptomatic nutritional support for 1 month and was discharged after improving.

## Discussion

ECD is a multisystem non-Langerhans histiocytosis that is most common in adult men (2, 4). Skeletal lesions occur in 95% of patients with characteristic multifocal osteosclerotic lesions of the long bones, and approximately one-third to one-half of patients present with pulmonary and cardiac disease, renal and skin involvement, and central nervous system symptoms (2, 3). The etiology and pathogenesis of ECD remain unclear, and controversy exists regarding whether ECD has a neoplastic or inflammatory nature (5). Cavalli et al. (1) reported that ECD histiocytes harbor oncogenic mutations in genes involved in the MAPK, signaling pathway (BRAF^V600E^ in more than half of the patients) and secrete abundant proinflammatory cytokines, such as interleukin (IL)-1, IL-6, and TNF-α, as well as chemokines. Based on these features, ECD is considered an inflammatory myeloid neoplasm (1, 6). In recent years, ECD was added to the classification of histiocytic and dendritic cell neoplasms (7). ECD with intestinal involvement is an uncommon condition, and the initial symptom of intestinal obstruction is even rarer, which has not been reported. The mechanism of intestinal obstruction might be maladaptive activation of immune programs in affected macrophages that caused the thickening of the intestinal wall, intestinal adhesion and intestinal stenosis in the patient reported in the present study. A history of syncope was considered related to previous moyamoya disease. Skin involvement presents with macula, and multiple systemic systems are involved leading to a poor nutritional status. Patients with ECD have various clinical presentations and radiologic findings, which makes the diagnosis challenging. Histological findings commonly include fibroinflammatory infiltrates containing foamy histiocytes (2). In images of IHC staining, ECD histiocytes are positive for CD68 and negative for S100 (2). Technetium-99 bone scans and PET/CT scans reveal increased uptake in affected areas (8).

Nearly all patients with ECD exhibit skeletal involvement, showing bilateral and symmetrical osteosclerosis changes on CT images (2, 3). The diagnosis of ECD depends on radiological and histopathological findings, and the exclusion of other diseases (9). The most important differential diagnosis is malignant tumor with multiple bone metastases, and the manifestations of bone metastases are mainly divided into osteoblast or lytic lesions according to the primary tumor. The lytic lesions are dominated by bone destruction and show hollow-like changes, while the images of osteoblast lesions show an increased bone density. In this case, the patient presented with a distinctive low-density lesion with a rosette of sclerosing edges that was distinct from bone metastases. In addition, the disease must be differentiated from Langerhans cell histiocytosis (LCH) before the establishment of the diagnosis, and a histopathological examination of ECD should be confirmed to be negative for Langerhans tissue cells. Factors associated with shorter survival of patients with ECD are central nervous system, retroperitoneal and pulmonary involvement (3, 9). The overall prognosis is poor because no clear radical treatment methods are available. BRAF and other MAPK pathway mutations are present in 50% of patients and have been targeted with BRAF inhibitors (2). Based on the understanding of ECD pathogenesis, Tomelleri et al. (6) and Berti et al. (10) reported that the efficacy of anakinra and tocilizumab was remarkable in patients with severe and difficult-to-treat disease manifestations such as cardiac involvement. Interferon and glucocorticoids have also been used as additional treatments to reduce symptoms (2, 3, 8).

## Data Availability Statement

The original contributions presented in the study are included in the article/supplementary material. Further inquiries can be directed to the corresponding author.

## Ethics Statement

Written informed consent was obtained from the individual(s) for the publication of any potentially identifiable images or data included in this article.

## Author Contributions

XZ, DZ, JY, DS, KL, GF, SJ, and WC conceived the idea for the article. XZ drafted the manuscript. WC approved the final version of the manuscript. All authors contributed to the article and approved the submitted version.

## Funding

This study was supported by the Project of State Key Laboratory of Radiation Medicine and Protection, Soochow University (No. GZK1202136, 1202009), “National Tutor System” Training Program for Health Youth Key Talents in Suzhou (Qngg2021006), China Nuclear Medicine 2021 “Nuclear Medical Science and Technology Innovation” (ZHYLYB2021006), Pre-research Foundation of Second Affiliated Hospital of Soochow University (SDFEYBS1904 and SDFEYQN1816), and Project of Nuclear Technology Medical Application Supported by Discipline Construction of Second Affiliated Hospital of Soochow University (XKTJ-HRC20210010).

## Conflict of Interest

The authors declare that the research was conducted in the absence of any commercial or financial relationships that could be construed as a potential conflict of interest.

## Publisher’s Note

All claims expressed in this article are solely those of the authors and do not necessarily represent those of their affiliated organizations, or those of the publisher, the editors and the reviewers. Any product that may be evaluated in this article, or claim that may be made by its manufacturer, is not guaranteed or endorsed by the publisher.
